# First Case of Esophagitis Dissecans Superficialis in an HIV Patient: A Case Report and Literature Review

**DOI:** 10.1155/2019/4616937

**Published:** 2019-07-02

**Authors:** Thaninee Prasoppokakorn, Palakorn Panarat

**Affiliations:** ^1^Department of Medicine, Faculty of Medicine, Chulalongkorn University, Bangkok, Thailand; ^2^Department of Medicine, Queen Savang Vadhana Memorial Hospital, Chonburi, Thailand

## Abstract

Esophagitis dissecans superficialis (EDS), a rare desquamative esophageal disease of uncertain etiology, is characterized by sloughing of fragments of esophageal mucosa. To the best of our knowledge, there has been no reported case of EDS in an HIV-infected patient. We report the first case of EDS in an adult HIV-infected male, who was hospitalized due to dysphagia. Esophagogastroscopy was performed, and the endoscopic findings together with the histopathologic findings of esophageal tissues were consistent with EDS. However, his symptom of dysphagia was not explained by EDS, but was the early symptoms of muscle-specific kinase (MuSK) myasthenia gravis (MG) that we finally diagnosed later by progression of the symptoms and electrophysiologic study. His symptoms had gradually improved after a course of intravenous immunoglobulin treatment. This is the first case of EDS and MuSK myasthenia gravis in an HIV-infected patient. A high index of suspicion of EDS should be made when taking care of the patients with desquamative or sloughing esophagitis especially with unknown etiology.

## 1. Introduction

Esophagitis dissecans superficialis (EDS), a very rare benign desquamative esophageal disease of uncertain etiology, is characterized by sloughing of large fragments of the esophageal squamous mucosa, followed by vomiting or regurgitation of the esophageal cast [1, 2]. To date, there have been a few reported cases of EDS in the published literature; hence, accurate characterization of clinical features, etiology, pathologic findings, and outcomes are lacking [2]. We report herein the first human immunodeficiency virus- (HIV-) infected case with EDS and review all EDS case reports in the English literature.

## 2. Case Report

**Figure 1:**
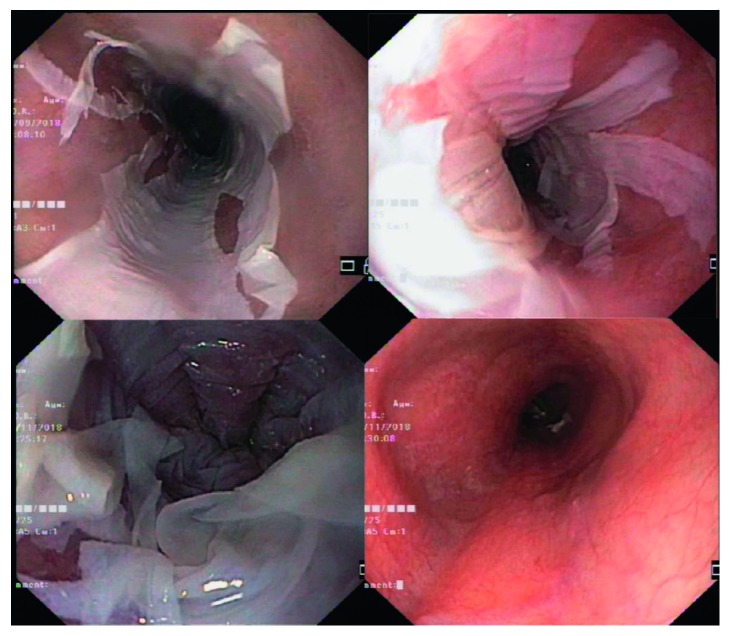
Esophagogastroscopy revealed diffuse pseudomembranes in the lower one-third esophagus.

**Figure 2:**
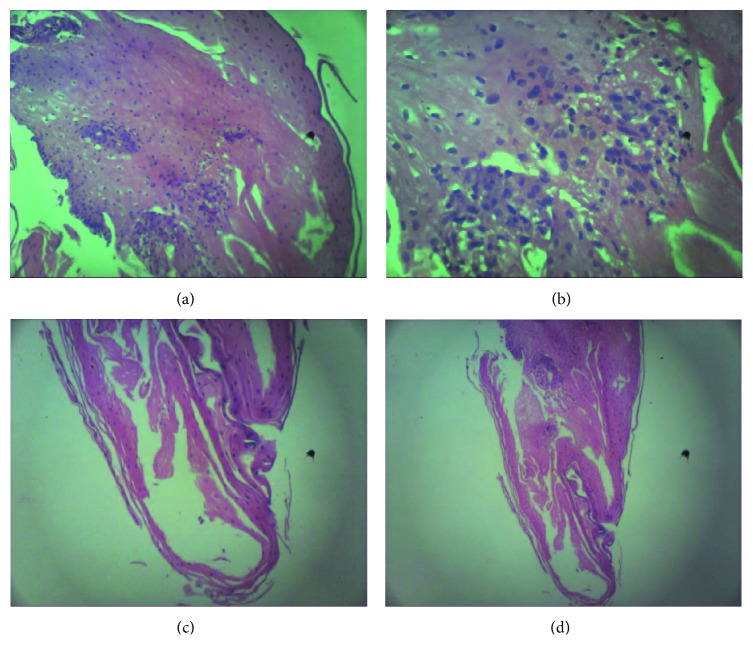
Pathologic findings of the lower esophagus revealed squamous cell hyperplasia (a), mild acute and chronic inflammatory infiltrations (b), a few small foci of splitting of the squamous epithelium (c), and a few intraepithelial cystic degenerations (d).

A 50-year-old male was hospitalized at Queen Savang Vadhana Memorial Hospital, Chonburi, Thailand, due to a 3-week course of oropharyngeal dysphagia. He had been diagnosed with HIV infection with a CD4 cell count of 64 cells/mL (8%) 7 years prior, when he presented with a 2-month history of chronic productive cough and significant weight loss. He received a combination treatment of tenofovir, emtricitabine, and efavirenz. Four months prior to the admission, he has achieved viral suppression with a CD4 count of 248 cells/mL (21%). Physical examination revealed neither oral candidiasis nor other intraoral lesions. Esophagogastroscopy was performed and revealed diffuse pseudomembranes in the lower one-third of the esophagus (Figure 1). The histopathologic findings of the biopsied esophageal mucosa exhibited mild acute and chronic esophagitis with focal squamous hyperplasia without demonstrated organisms. A presumptive diagnosis of candidal esophagitis was made, and fluconazole of 200 mg/day was prescribed to the patient. No clinical improvement was observed at 3 weeks after the start of antifungal treatment. Fluconazole dose was increased to 400 mg/day. Three weeks after high-dose fluconazole treatment, he was rehospitalized due to worsening condition and development of hypovolemic hyponatremia. Esophagogastroscopy was done once again, which revealed circumferential white exudates with focal areas of erosions at the lower one-third of the esophagus without luminal stenosis or obstruction. The pathologic findings exhibited foci of splitting of the squamous epithelium with few intraepithelial cystic degenerations, no parakeratosis, no basal cell hyperplasia, mild acute and chronic inflammatory infiltrates with focal squamous cell hyperplasia, and normal underlying mucosa, which were similar to the previous findings (Figure 2). A diagnosis of EDS was made, and omeprazole of 80 mg/day was given to the patient. The patient's symptoms gradually improved after 2 weeks of treatment. During hospitalization, he developed binocular diplopia, proximal muscle weakness, and ventilatory failure requiring ventilator support. Myasthenia gravis was suspected. The diagnosis was confirmed by an electrophysiologic study and positive result of muscle-specific tyrosine kinase (muscle-specific kinase, MuSK). The patient gradually improved after a course of intravenous immunoglobulin treatment.

## 3. Discussion

To the best of our knowledge, this is the first case of EDS in an HIV-infected patient. EDS is a rare self-limited desquamative esophagitis of unknown etiology, usually affects adults after the age of 50 [1]. It is likely underestimated and frequently misdiagnosed as in our case. The diagnostic criteria of EDS have not been established yet; however, the endoscopic and pathologic criteria were recently proposed by Hart et al. including the presence of all of the following features: (1) the strip(s) of sloughed esophageal mucosa of >2 cm in length, (2) the normal underlying mucosa, and (3) the lack of ulcerations or friability of immediately adjacent mucosa [2].

The etiology of EDS is unknown. There have been many reports demonstrating the association of EDS with (1) drugs including nonsteroidal anti-inflammatory drugs [1, 3, 4], bisphosphonates [5], potassium chloride [6], and clindamycin [7]; (2) celiac disease; and (3) autoimmune bullous dermatoses [6]. To date, there has been no report about the association of EDS and HIV infection, as in our case. Interestingly, due to lack of similar cases, the association between antiretroviral drugs and EDS required further studies. Similarly, the association between EDS and other immunodeficiency conditions has not been described. As a result, it is uncertain whether the HIV virus itself plays a role in the development of EDS. Furthermore, the association between EDS and MG also has never been reported. The presenting symptoms vary in different reports including dysphagia, heartburn, regurgitation, dyspepsia, upper gastrointestinal bleeding, anemia, and weight loss [2]. EDS is a benign condition that can resolve spontaneously or with the treatment with a proton-pump inhibitor (PPI) [1, 4]. Our patient had protracted course of dysphagia for 6 weeks before clinical improvement with PPI treatment.

In addition, we review all 30 adult patients (including our case) with EDS [1, 4, 5, 7–11] (Table 1). There were 15 males and 15 females with the mean age of 65.8 ± 14.9 (range: 42–88) years. Our case is the first case with HIV infection. Regarding the race, there were 1 Asian (our case) and 28 Caucasians. The presenting symptoms included dysphagia (12 patients, 40.0%), upper gastrointestinal bleeding (4, 13.3%), epigastric pain (4, 13.3%), heartburn (3, 10.0%), weight loss (3, 10.0%), anemia (2, 6.7%), regurgitation (1, 3.3%), and atypical chest pain (1, 3.3%). Of 30 patients undergoing endoscopy, 21 (70.0%) patients had the characteristic features of EDS (the distinct features of denuded mucosal lining or peeling mucosa of the distal esophagus). Of 30 patients, the histology of the esophageal biopsy exhibited the sloughing of superficial layer of epithelium (30 patients, 100.0%), the presence of eosinophils on the surface (4, 13.3%), parakeratosis (17, 56.7%), and normal underlying mucosa (2, 6.7%). Of 30 patients with available data, all with (21 patients, 70.0%) and without PPI treatment (9, 30.0%) had good outcome with both clinical and endoscopic improvement.

## 4. Conclusion

To the best of our knowledge, this is the first case of EDS in an HIV-infected patient. Due to lack of similar cases, the association between immunodeficiency conditions including antiretroviral drugs and EDS required further studies. EDS is a rare self-limited desquamative esophagitis of unknown etiology, which is diagnosed by excluding all other possible causes of oropharyngeal dysphagia. A high index of suspicion of EDS should be made when taking care of patients with desquamative or sloughing esophagitis especially with unknown etiology and/or no response to antifungal treatment.

## Figures and Tables

**Table 1:** A summary of all 30 cases with esophagitis dissecans superficialis (EDS).

Patient	Gender/age (year)	Race	Presentation	Diagnosis and associated condition	Endoscopic finding	Histology	Treatment	Outcome
1 [8]	F/81	Canadian	Chronic intermittent dysphagia	No significance	A thin white membrane lifting from mucosa	Mild candidal esophagitis; splitting of squamous mucosa at suprabasal and superficial layers; focal lymphocytic infiltration at deepest surfaces and diffuse parakeratosis	Double dose of PPI	Improvement
2 [9]	M/51	NA	Intermittent heartburn, anorexia, and epigastric pain without dysphagia	NSAID use	A thin white membrane lifting from mucosa with necrotic zones located on the lower esophagus	Detached superficial strips of squamous epithelium; prominent parakeratosis; acute inflammation and spongiosis in deep layers of squamous epithelium	Double dose of PPI	Endoscopic resolution 2 weeks after treatment
3 [10]	F/76	Belgian	Weight loss and nausea	COPD, CABG, hypertension	Fibrotic and sclerotic aspect of distal third of the esophagus	Sloughing of superficial layer of esophageal epithelium; presence of eosinophils on surface	No	Improvement
4 [10]	F/70	Belgian	Epigastric pain	Gastric bypass	White pseudomembranes of mid and distal third of the esophagus	Sloughing of superficial layer of esophageal epithelium; presence of PMN on surface separating it from basal layer	No	NA
5 [10]	F/71	Belgian	Epigastric pain	Arthrodesis L5-S1	Elevated lesion of distal third of the esophagus	Sloughing of superficial layer of esophageal epithelium; presence of eosinophils on surface	No	Improvement
6 [10]	F/47	Belgian	Dysphagia	Gastrectomy with eso-jejunal anastomosis, hypothyroidism, autoimmune pancreatitis	Sloughed esophageal mucosa in the esophagus	Sloughing of superficial layer of esophageal epithelium; presence of a few bacterial colonies on surface	No	Improvement
7 [10]	F/77	Belgian	Epigastric pain	Hypertension, hypercholesterolemia, hepatitis C, OSA	Sloughing of esophageal mucosa in the distal two-third esophagus	Sloughing of superficial layer of esophageal epithelium separated from normal basal layer	No	Improvement
8 [10]	M/86	Belgian	Atypical chest pain	Hypertension, hyperuricemia	Grade A reflux esophagitis	Sloughing of superficial layer of esophageal epithelium separated from normal basal layer	No	NA
9 [10]	F/83	Belgian	Dysphagia	Hypertension hypothyroidism, Alzheimer disease, DVT	Suspicion of an esophageal tumor from 16 to 24 cm of dental arch, circumferential from 18 to 20 cm	Sloughing of superficial layer of esophageal epithelium, presence of PMN on surface separating it from basal layer	No	Improvement
10 [7]	F/42	Portuguese	Dysphagia	Type 1 diabetes mellitus, clindamycin treatment for diabetic foot	Sloughing reddish membranes in the distal esophagus, adjacent to intact easily removed mucosa	Squamous epithelium detached from underlying basal layer; preservation of remaining layers; mild parakeratosis; severe acute inflammation with some eosinophils	Double dose of PPI	Improvement
11 [5]	F/59	USA	Dysphagia and odynophagia	Hypersensitivity pneumonitis, bisphosphonate	Sloughing whitish membranes that were easily removed, adjacent to intact healthy mucosa	Parakeratosis and desquamation of epithelial layer	Bisphosphonate discontinuation	Improvement
12 [4]	M/46	Caucasian	Nausea, vomiting, regurgitation	Hypertension, NSAID use	Distinct features of denuded mucosal lining, stripped off mucosa, long mucosal breaks, linear furrows, and vertical fissures	Fragments of superficial epithelium, parakeratosis; coagulative necrosis with epithelial sloughing	Double dose of PPI; NSAID discontinuation	Improvement
13 [4]	F/42	Caucasian	Dysphagia, regurgitation	GERD	Distinct features of denuded mucosal lining, stripped off mucosa, long mucosal breaks, linear furrows, and vertical fissures	Fragments of superficial epithelium, parakeratosis; coagulative necrosis with epithelial sloughing	Single dose of PPI	Improvement
14 [4]	F/79	African American	Dysphagia, nausea, vomiting	GERD, bullous pemphigoid, diabetes	Distinct features of denuded mucosal lining, stripped off mucosa, long mucosal breaks, linear furrows, and vertical fissures	Fragments of superficial epithelium, parakeratosis; coagulative necrosis with epithelial sloughing	Single dose of PPI	Improvement
15 [4]	F/46	African American	Dysphagia, dyspepsia	GERD, hypertension, diabetes	Distinct features of denuded mucosal lining, stripped off mucosa, long mucosal breaks, linear furrows, vertical fissures	Fragments of superficial epithelium, parakeratosis; coagulative necrosis with epithelial sloughing	Double dose of PPI	Improvement
16 [4]	F/55	Hispanic	Heartburn	GERD, hypertension, NSAID use	Distinct features of denuded mucosal lining, stripped off mucosa, long mucosal breaks, linear furrows, and vertical fissures	Fragments of superficial epithelium, parakeratosis; coagulative necrosis with epithelial sloughing	Single dose of PPI; NSAID discontinuation	Improvement
17 [1]	M/61	White	Dysphagia	NA	Diffuse erythema, patches of peeling mucosa in the mid to distal esophagus	Parakeratosis; fragments of necrotic epithelium with minimal inflammation; intraepithelial splitting at varying degrees above basal layer	Double dose of PPI	Improvement
18 [1]	M/81	African American	UGIB	NA	Multiple patches of peeling mucosa	Parakeratosis; fragments of necrotic epithelium with minimal inflammation; intraepithelial splitting at varying degrees above basal layer	Double dose of PPI	Improvement
19 [1]	M/77	African American	Weight loss	NA	Distal 2 cm patch of peeling mucosa	Parakeratosis; fragments of necrotic epithelium with minimal inflammation; intraepithelial splitting at varying degrees above basal layer	Double dose of PPI	NA
20 [1]	M/65	White	Dysphagia	NA	Grade 4 esophagitis	Parakeratosis; fragments of necrotic epithelium with minimal inflammation; intraepithelial splitting at varying degrees above basal layer	Double dose of PPI	Improvement
21 [1]	M/60	African American	UGIB	NA	Grade 3 esophagitis from the mid to distal esophagus	Parakeratosis; fragments of necrotic epithelium with minimal inflammation; intraepithelial splitting at varying degrees above basal layer	Double dose of PPI	Improvement
22 [1]	M/57	African American	Epigastric pain	NA	Distal multiple patches of peeling mucosa	Parakeratosis; fragments of necrotic epithelium with minimal inflammation; intraepithelial splitting at varying degrees above basal layer	Double dose of PPI	NA
23 [1]	F/49	White	Heartburn	NA	Mid to distal grade 3 esophagitis, achalasia	Parakeratosis; fragments of necrotic epithelium with minimal inflammation; intraepithelial splitting at varying degrees above basal layer	Double dose of PPI	Improvement
24 [1]	M/65	White	Anemia	NA	Mid to distal grade 4 esophagitis	Parakeratosis; fragments of necrotic epithelium with minimal inflammation; intraepithelial splitting at varying degrees above basal layer	Double dose of PPI	NA
25 [1]	M/80	White	Iron deficiency anemia	NA	Mid to distal grade 4 esophagitis	Parakeratosis; fragments of necrotic epithelium with minimal inflammation; intraepithelial splitting at varying degrees above basal layer	Double dose of PPI	NA
26 [1]	M/84	White	UGIB	NA	Sloughing of entire esophagus	Parakeratosis; fragments of necrotic epithelium with minimal inflammation; intraepithelial splitting at varying degrees above basal layer	Double dose of PPI	NA
27 [1]	M/63	White	Dysphagia	NA	Multiple patches of peeling mucosa in the mid to distal esophagus	Parakeratosis; fragments of necrotic epithelium with minimal inflammation; intraepithelial splitting at varying degrees above basal layer	Double dose of PPI	NA
28 [1]	M/84	White	Weight loss	NA	Distal adherent yellow patches of peeling mucosa	Parakeratosis; fragments of necrotic epithelium with minimal inflammation; intraepithelial splitting at varying degrees above basal layer	Double dose of PPI	NA
29 [11]	F/88	USA	UGIB	Atrial fibrillation, mitral regurgitation, post-cardiac arrest syndrome	White, linear, desquamating plaques in the distal esophagus	Sloughing of superficial layer of esophageal epithelium; presence of eosinophils on surface; parakeratosis	No	Improvement
30 (our case)	M/50	Thai	Dysphagia	HIV infection, myasthenia gravis	Circumferential white exudates with focal areas of erosions at the lower one-third esophagus without luminal stenosis or obstruction	Foci of splitting of the squamous epithelium with few intraepithelial cystic degeneration; no parakeratosis; no basal cell hyperplasia; mild acute and chronic inflammatory infiltrates with focal squamous cell hyperplasia; and normal underlying mucosa	Double dose of PPI	Improvement

COPD: chronic obstructive pulmonary disease; CABG: coronary artery bypass graft; OSA: obstructive sleep apnea; DVT: deep vein thrombosis; UGIB: upper gastrointestinal bleeding; PMN: polymorphonuclear cell; PPI: proton-pump inhibitor; NSAID: nonsteroidal anti-inflammatory drug.
